# Alexithymia and the Processing of Emotional Facial Expressions (EFEs): Systematic Review, Unanswered Questions and Further Perspectives

**DOI:** 10.1371/journal.pone.0042429

**Published:** 2012-08-23

**Authors:** Delphine Grynberg, Betty Chang, Olivier Corneille, Pierre Maurage, Nicolas Vermeulen, Sylvie Berthoz, Olivier Luminet

**Affiliations:** 1 Research Institute for Psychological Sciences, Université catholique de Louvain, Louvain-la-Neuve, Belgium; 2 Inserm U669, Univ.Paris-Descartes and Paris-Sud and Institut Mutualiste Montsouris, Paris, France; 3 Research Institute for Psychological Sciences, Université catholique de Louvain, Louvain-la-Neuve, Belgium; Institute of Psychiatry at the Federal University of Rio de Janeiro, Brazil

## Abstract

Alexithymia is characterized by difficulties in identifying, differentiating and describing feelings. A high prevalence of alexithymia has often been observed in clinical disorders characterized by low social functioning. This review aims to assess the association between alexithymia and the ability to decode emotional facial expressions (EFEs) within clinical and healthy populations. More precisely, this review has four main objectives: (1) to assess if alexithymia is a better predictor of the ability to decode EFEs than the diagnosis of clinical disorder; (2) to assess the influence of comorbid factors (depression and anxiety disorder) on the ability to decode EFE; (3) to investigate if deficits in decoding EFEs are specific to some levels of processing or task types; (4) to investigate if the deficits are specific to particular EFEs. Twenty four studies (behavioural and neuroimaging) were identified through a computerized literature search of Psycinfo, PubMed, and Web of Science databases from 1990 to 2010. Data on methodology, clinical characteristics, and possible confounds were analyzed. The review revealed that: (1) alexithymia is associated with deficits in labelling EFEs among clinical disorders, (2) the level of depression and anxiety partially account for the decoding deficits, (3) alexithymia is associated with reduced perceptual abilities, and is likely to be associated with impaired semantic representations of emotional concepts, and (4) alexithymia is associated with neither specific EFEs nor a specific valence. These studies are discussed with respect to processes involved in the recognition of EFEs. Future directions for research on emotion perception are also discussed.

## Introduction

Alexithymia is a multifaceted personality construct characterized by an impaired ability to reflect on and regulate one's own emotions. This construct was initially introduced by Nemiah, Freyberger, and Sifnéos [1] to describe clinical patients with psychosomatic problems who experience difficulties describing their emotions and who exhibit impoverished mental representations of their emotional states. Yet, since then, alexithymia has been documented in a variety of psychiatric illnesses [2]. Sifnéos [3] described alexithymic individuals as presenting: “*an impoverishment of fantasy life, a constriction of emotional functioning, and a difficulty in interpersonal relations; a striking inability to find appropriate words to describe emotions and a tendency to describe endless situational details or symptoms instead of feelings; a preference for using action to avoid conflicting situations; rigid postures, including sitting in such a way as to give the impression that the person is frozen into one position” (p.67)*. Here, Sifnéos used the word “feeling” to describe the subjective fantasies and thoughts associated with emotions. At an intrapersonal level, alexithymia is commonly characterized by difficulties in identifying feelings (DIF), difficulties in describing feelings (DDF) and by an externally-oriented thinking style (EOT). The first two factors refer to a poor emotional awareness and the third factor refers to a specific tendency to deal with superficial themes and to avoid affective thinking [4]. Oddly enough, although Sifnéos and Nemiah's description encompassed deficits in the interpersonal domain, very few studies attempted to objectively investigate whether these deficits are part of the core alexithymia features.

Bagby and Taylor were the first to further describe the core features of alexithymia and stated that alexithymic characteristics “*reflect deficits both in the cognitive-experiential domain of emotion response system and at the level of interpersonal regulation of emotion. […] Lacking knowledge of their own emotional experiences, alexithymic individuals cannot readily imagine themselves in another person's situation and are consequently unempathetic and ineffective in modulating the emotional states of others*” ([5], p 30–31). Since then, alexithymia has been repeatedly related to interpersonal deficits such as empathy impairments (e.g., [6], [7], [8], [9]) and it is only recently that this construct was investigated by researchers in the field of social neuroscience [10], [11]. There is now a body of psychometric, behavioural and neuroimaging studies that attempts to capture and specify the processes that may account for the observed associations between the inability to monitor and self-regulate emotions and compromised social understanding and interactions (see [12]).

Since the 1970s, among the advances that have occurred in alexithymia research, the alexithymia construct has benefited in particular from the increasing knowledge of the underlying mechanisms of normal and pathological emotion recognition and understanding (using verbal, facial, and body cues). Importantly, emotional expressions, through their signal functions, are suggested to promote the forging of relationships among individuals within a social group [13], [14]. The accurate perception of emotional expressions allows people to adjust to the emotional states and behaviours of others [15], [16]. Among these, the processing of emotional facial expressions (i.e., EFEs) is central to effective social interactions and interpersonal understanding [17]. The recognition of EFEs is also fundamental to social adjustment, and more precisely, to the experience of empathy (e.g., [18], [19]). Empathy requires the identification of others' emotional expressions and the corresponding regulation of one's own emotional state, two processes that are supposed to be impaired in alexithymia [20]. As summed up by Atkinson et al., “*recognition of another's emotional expression likely involves processes that link the perceptual properties of the stimulus to various knowledge structures, such as the specific emotion concept, the lexical label for that emotion, and the perception of the emotional response (or a central representation thereof) that the stimulus triggers in the observer*” ([21], p 60).

Alexithymia is a transdiagnostic deficit, meaning that it is considered as a common deficit of several disorders. The study of alexithymia may offer new perspectives in determining the pathological mechanisms and vulnerability factors leading to clinical disorders associated with poor social skills and with EFEs processing deficits, such as autism spectrum condition, eating disorders and social anxiety disorders [12]. Recent theories suggest that rather than using separated categories to classify mental or personality disorders, one's can describe these disorders in terms of cognitive and affective processes [22]. These theories propose a transversal approach rather than specific diagnostic categories and suggest that beyond these categories, disorders share common cognitive (e.g., rumination) and/or affective symptoms (e.g., emotion regulation deficits). They are suggested to be involved in the onset and maintenance of these disorders [22]. For instance, it has been shown that rumination, which is common to depression and anxiety, predicts the severity and duration of depression [23]. Because alexithymia is prevalent in disorders characterized by EFEs processing deficits, alexithymia might be a better predictor of the ability to decode EFEs than the diagnosis per se. Moreover, understanding the links between alexithymia and the processing of EFEs could help us to tailor therapeutic approaches to better address the poor prosocial skills associated with alexithymia. Indeed, there is growing evidence that training people with schizophrenia and autism spectrum condition to decode EFEs can improve their social relationships (e.g., [24], [25], [26]).

Here we review studies that investigated the associations between alexithymia and EFE processing in order to clarify the existing relations between the two factors and to further examine whether alexithymia is a core factor that could account for common emotion recognition peculiarities that have been described in several psychiatric disorders. Studies on five types of clinical disorders associated with abnormal social adaptation and emotional stimuli processing and with high alexithymia scores have been found: eating disorders, autism spectrum disorders, drug abusers (alcohol and other substance abuse), patients with panic disorder and patients with somatoform disorders. Results that were obtained in normal individuals with high alexithymia scores were also included to complement the studies with abnormal/disordered populations. Such studies should give support to the findings observed among clinical populations. We argue that both alexithymia and deficits in EFEs decoding are exacerbated among clinical relative to healthy populations. Studies in normal individuals might thus corroborate the findings among clinical populations but might also give additional information as no study has investigated in clinical populations the association between alexithymia and neural correlates of EFEs processing. We will indeed discuss neuroimaging studies showing that brain structures associated with emotional evaluation are abnormally activated when healthy people with high alexithymia scores process EFEs.

The disorders that encompass a large proportion of high alexithymia scorers also have a high comorbidity with depressive and anxiety disorders. In addition, normal individuals with high alexithymia scores also display high levels of self-reported depression and anxiety, which have been found to bias negative emotional information processing (e.g., [27], [28], [29]). Therefore, it is important to control for these affective dimensions when investigating how alexithymia may be associated with impairments in processing EFEs in both clinical and healthy populations. Only the most recent studies have taken into account the potential confounding of current dysphoric affect and anxiety with alexithymia.

The present review includes an exhaustive overview of studies that have emphasized EFE processing deficits in alexithymia (see Table 1 for a summary). In order to assess if alexithymia scores predict EFE processing deficits, we selected studies that tested either clinical (restricted to the five groups mentioned above) or healthy populations. We also included neuroimaging studies in order to examine the neural basis of EFE processing deficits in alexithymia. Before proceeding to the review proper, we first describe the objectives and the selection method.

**Table 1:** Summary of the characteristics of the studies reviewed.

	Task	EFE Database	EFE	N^ber^ of stimuli	Nature of stimuli	Duration	Population	Sample size	Alexithymia Scale	Categorical Approach	Controlled dimensions	Behavioural Deficits	Type of Brain Analysis	Brain activity
**Clinical Populations- Behavioural studies**														
**Mann et al. ** [**54**]	Labelling	PFA	Sadness, Happiness, Fear, Anger, Disgust, Surprise	5	Static	10 sec	Substance Abusers+Control	80	TAS-26 (total score)	No	None	None	**-**	-
**Galderisi et al. ** [**55**]	Labelling	Schematic faces	Ambiguous faces Anger, Disgust, Fear, Happiness, Sadness, Rejection, Acceptance	1	Static	Undefined	Panic Disorder+Control	54	TAS-20 (total score and subscales)	No	None	None		
**Kessler et al. ** [**56**]	Labelling	JACFEE	Sadness, Happiness, Fear, Anger, Disgust, Surprise	7	Static	300 mesc	Eating disorder+Control	157	TAS-26 (total score)	No	None	None	-	-

	Task	EFE Database	EFE	N^ber^ of stimuli	Nature of stimuli	Duration	Population	Sample size	Alexithymia Scale	Categorical Approach	Controlled dimensions	Behavioural Deficits	Type of Brain Analysis	Brain activity
**Pedrosa Gil et al. ** [**57**]	Labelling	JACFEE	Sadness, Happiness, Fear, Anger, Disgust, Surprise	7	Static	300 mesc	Somatoform disorder+Control	40	TAS-26 (total score)	No	None	Alexithymia negatively correlated with total score labelling	-	-
**Kätsyri et al. ** [**59**]	Labelling	Unstan-dardized	Anger, Disgust, Fear, Happiness	4	Static and Dynamic	2 sec (Static)3 sec (Dynamic)	Asperger syndrome+Control	40	TAS-20 (total score and subscales)	No	Schizophrenia, Obsessive-compulsory disorders, severe depression or learning difficulties, prosopagnosia	Index of degradation with TAS-20 total and all subscales scores	-	-

	Task	EFE Database	EFE	N^ber^ of stimuli	Nature of stimuli	Duration	Population	Sample size	Alexithymia Scale	Categorical Approach	Controlled dimensions	Behavioural Deficits	Type of Brain Analysis	Brain activity
**Ridout et al. ** [**60**]	Labelling	TASIT	Sadness, Happiness, Fear, Anger, Disgust, Surprise, Neutral	4	Dynamic	15–60 sec	Healthy with low and high vulnerability for disordered eating	45	TAS-20 (total score)	No	Anxiety, Depression	Alexithymia negatively correlated with total score labelling	-	-
**Clinical population – Neuroimaging studies**														
**Pollatos et al. ** [**62**]	Labelling	KDEF	Sadness, Happiness, Fear, Anger, Disgust, Neutral	40		2 sec	Anorexia Nervosa+Control	30	TAS-20 (total score and subscales)	No	None	DIF and DDF negatively correlate with neutral faces	ERP	None

	Task	EFE Database	EFE	N^ber^ of stimuli	Nature of stimuli	Duration	Population	Sample size	Alexithymia Scale	Categorical Approach	Controlled dimensions	Behavioural Deficits	Type of Brain Analysis	Brain activity
**Healthy Populations- Behavioural studies**														
**Prkachin et al. (study 2) ** [**65**]	Labelling and Intensity rating	PFA	Sadness, Happiness, Fear, Anger, Disgust, Surprise	6	Static	Undefined	Healthy	43	TAS-20 (total score and subscales)	No	None	No labelling deficits. Negative correlations between EOT and DIF and fear intensity rating	**-**	-
**Mann et al. ** [**66**]	Labelling	PFA	Sadness, Happiness, Fear, Anger, Disgust, Surprise	5	Static	10 sec	Healthy	62	TAS-26 (Total score)	22 LA : TAS-26< or = 4919 MA : 50<TAS-26<5721 HA : TAS-26> or = 58	None	HA<LA labelling all emotions (Total score) and specifically sadness	**-**	-

	Task	EFE Database	EFE	N^ber^ of stimuli	Nature of stimuli	Duration	Population	Sample size	Alexithymia Scale	Categorical Approach	Controlled dimensions	Behavioural Deficits	Type of Brain Analysis	Brain activity
**Jessimer and Markham ** [**67**]	Labelling	PFA	Sadness, Happiness, Fear, Anger, Disgust, Surprise	2	Static	Undefined	Healthy	36	TAS-20 (DIF and DDF)	18 LA : <10th percentile18 HA : >90th percentile	None	HA<LA labelling all emotions	-	-
**Montebarocci et al. ** [**68**]	Labelling	PFA	Sadness, Happiness, Fear, Anger, Disgust, Surprise, Neutral	6	Static	10 sec	Healthy	35	TAS-20 (total score)	18 LA: TAS-20< = 5117 HA: TAS-20> = 61	Depression, anxiety, verbal IQ	HA<LA labelling all emotions and neutral	-	
**Swart et al. ** [**69**]	Labelling	PFA	Sadness, Happiness, Fear, Anger, Disgust, Surprise, Contempt	2	Static	15 msec	Healthy	34	BVAQ (verbalizing)	17 LA<Mean−1.5 SD17 HA>Mean+1.5 SD	None	HA<LA labelling total score	-	-

	Task	EFE Database	EFE	N^ber^ of stimuli	Nature of stimuli	Duration	Population	Sample size	Alexithymia Scale	Categorical Approach	Controlled dimensions	Behavioural Deficits	Type of Brain Analysis	Brain activity
**Pandey & Mandal ** [**70**]	Labelling	Unstandar-dized	Sadness, Happiness, Fear, Anger, Disgust, Surprise	2	Static	10 sec	Healthy	24	TAS-20 (total score)	Cutoff scores of 1.5 SD above and below the mean12 LA : TAS-20< or = 3312 HA : TAS-20> or = 68	Psychiatric, neurologic, chronic illness and manifest speech disturbance	None	-	-
**Lane et al. ** [**71**]	LabellingMatching	Unstandardized	Sadness, Happiness, Fear, Anger, Disgust, Surprise, Neutral	6	Static	Undefined	Healthy	380	TAS-20 (total score)	No	Anxiety	Negative correlation between TAS-20 total and total score (emotions and neutral expression)	-	**-**
**Lane et al. ** [**72**]	LabellingMatching	Unstandar-dized	Sadness, Happiness, Fear, Anger, Disgust, Surprise, Neutral	6	Static	Undefined	Healthy	379	TAS-20 (total score	1. Cutoff259 LA : TAS-20≤ 5170 MA: 51<TAS-20<6050 HA: TAS-20≥612. Cutoff scores of 1 SD above	None	HA<LA for all emotions+Neutral (with both cutoffs)	-	-

	Task	EFE Database	EFE	N^ber^ of stimuli	Nature of stimuli	Duration	Population	Sample size	Alexithymia Scale	Categorical Approach	Controlled dimensions	Behavioural Deficits	Type of Brain Analysis	Brain activity
										and below the mean67 LA : TAS-≤34241 MA : 35<TAS-20<57 71 HA : TAS-20≥58				
**Prkachin et al. (study 1) ** [**65**]	Matching	PFA	Sadness, Happiness, Fear, Anger, Disgust, Surprise	6	Static	33 msec	Healthy	128	1. TAS-20 (total score) 2. TAS-20 (total score and subscales)	1. Cutoff of Taylor et al. (1997)60 LA : TAS-20≤5147 MA : 51<TAS-20<6121 HA : TAS-20≥612. No	None	1. HA<LA matching sad, anger, fear faces2. Negative correlation between EOT and total scores with all basic emotions	**-**	
**Parker et al. ** [**73**]	Detection	PFA	Anger, Sadness, Fear	50	Static	1 sec and 3 sec	Healthy	146	TAS-20 (total score and subscales)	No	Positive and Negative affectivity	Negative correlation between DDF and total score of detection	-	-

	Task	EFE Database	EFE	N^ber^ of stimuli	Nature of stimuli	Duration	Population	Sample size	Alexithymia Scale	Categorical Approach	Controlled dimensions	Behavioural Deficits	Type of Brain Analysis	Brain activity
**Healthy Populations- Neuroimaging studies**														
**Eichmann et al. ** [**74**]	Masked presentation	PFA	Sadness, Happiness, Neutral	20	Static	33 mesc	Healthy	22	TAS-20 (total score and subscales)	No	None	None	Method: ROI analyses.	Negative correlation between TAS-20 total and DIF and activation in the fusiform gyrus for Sadness
**Kügel et al. ** [**75**]	Masked presentation	PFA	Sadness, Happiness Neutral	20	Static	33 mesc	Healthy	21	TAS-20 (total score and subscales)	No	Depression, Anxiety	None	Method: ROI analyses.	(after controlling for BDI and STAI) Negative correlation between TAS-20 total and DIF and activation in the Amygdala for Sadness
**Reker et al. ** [**76**]	Masked presentation	PFA	Sadness, Happiness, Neutral	20	Static	33 mesc	Healthy	33	TAS-20 (total score)	No	Depression, Anxiety	None	Method: whole brain analysis; ROI analyses.	For Sadness (after controlling for BDI and STAI), negative correlation between total score and the left amygdala, inferior frontal gyrus, superior temporal gyrus, fusiform gyrus,

	Task	EFE Database	EFE	N^ber^ of stimuli	Nature of stimuli	Duration	Population	Sample size	Alexithymia Scale	Categorical Approach	Controlled dimensions	Behavioural Deficits	Type of Brain Analysis	Brain activity
														middle occipital and parahippocampal gyrus, bilateral insula.Negative correlations between DIF, DDF and left amygdala For Happiness (after controlling for BDI and STAI): Negative correlation total score and bilateral inferior frontal gyrus, insula, and superior temporal gyrus, right superior parietal lobule, precuneus, and parahippocampal gyrus, and the left cuneus, claustrum, postcentral and middle occipital gyrus

	Task	EFE Database	EFE	N^ber^ of stimuli	Nature of stimuli	Duration	Population	Sample size	Alexithymia Scale	Categorical Approach	Controlled dimensions	Behavioural Deficits	Type of Brain Analysis	Brain activity
**Duan et al. ** [**77**]	Masked presentation	Chinese Affective Picture System	Surprise, Happy, Neutral	120	Static	33 mesc	Healthy	18	TAS-20 (total score)	No	None	None	Method: Whole brain analysis.	Negative correlation between DIF and activation in the left fusiform gyrus, the right parahippocampal gyrus, and the right superior temporal gyrus.
**Kano et al. ** [**88**]	Gender decision	ATR	Anger, Sadness, Happiness, Neutral	60 for each emotion20 for neutral	Static	3 sec	Healthy	24	TAS-20 (total score)	12 LA : TAS-20<51 12 HA: TAS-20>61	None	HA rated sad faces as disgust	Method: Whole brain analysis	- Angry vs. Neutral : LA>HA: right insula, right ACC, right superior temporal gyrus HA>LA: left temporal sub-lobar - Sad vs. Neutral : LA>HA: right insula - Happy vs. Neutral : LA>HA: right mPFC (BA10), right insula - Negative correlations between brain activity (Angry vs. baseline & Sad vs. baseline) and TAS20 scores in right orbitofrontal cortex and right IFG

	Task	EFE Database	EFE	N^ber^ of stimuli	Nature of stimuli	Duration	Population	Sample size	Alexithymia Scale	Categorical Approach	Controlled dimensions	Behavioural Deficits	Type of Brain Analysis	Brain activity
**Mériau et al. ** [**92**]	Gender decision Labelling	PFA	Anger, Fear	72	Static	3.75 sec	Healthy	23	TAS-20 (total score)	No (but connectivity: median split)	Positive and negative affectivity Anxiety	None	Method: Whole brain analysis; ROI analyses; connectivity between dACC and other regions	Positive correlation between TAS-20 total and left dorsal ACC during gender decision. HA : higher connectivity between left dorsal ACC and amygdala; LA : higher connectivity between left dorsal ACC and prefrontal cortex
**Heinzel et al. ** [**93**]	Passive viewing	PFA	Fear, Happiness, Neutral	50	Static	4 sec	Healthy	60	TAS-20 (total score)	30 LA: <33th percentile: Mean = 33.3230 HA: >66th percentileMean = 59.06	Depression	None	Method Whole brain and ROI for Amygdala and ACC.	HA: stronger ACC activation for Fear, Happiness
**Vermeulen et al. ** [**95**]	Categorical perception	PFA	Anger, Disgust	8	Static	500 msec	Healthy	20	TAS-20 (total score)	Cutoff scores of 1 Standard Deviation above and below the mean10 LA : TAS-20≤34.7010 HA : TAS-20≥63.40	Positive and negative affectivity Anxiety, Depression	None	Event-related Potential	N2b component delayed in response to angerP3a componentdelayed for disgust and anger

## Objectives

Among the studies that investigated the relationship between alexithymia and EFE processing in clinical populations, it is not always possible to disentangle the effects of the diagnostic per se from the effects of alexithymia.

Objective 1 of this review will be to investigate if alexithymia is a better predictor of performance differences than an individual's clinical status (i.e., whether they have a clinical disorder or not). We will also examine whether the reported deficits observed in clinical groups may be extended to non-clinical populations, and we will discuss the few studies that adjusted for the main effect of alexithymia.

Objective 2 will be to assess if other affective dimensions, in particular depression and anxiety, could account for some of the relationship between alexithymia and EFE processing.

Objective 3 will be to examine if the reported deficits in alexithymia concerned a specific type of processing (e.g., perceptual proprieties of EFEs; emotion concepts; lexical labels) or if these deficits appeared only under a specific condition (processing of emotional vs. non-emotional information on EFEs).

Finally, Objective 4 will be to provide a clear overview of the deficits in high alexithymia scorers, in particular whether they are specific to the processing of a particular valence (positive, negative, or neutral) of EFEs and/or of particular EFEs. Here, in order to keep the label usually found in the literature (and in the studies included in the present review), we will use the term “neutral” to refer to expressions in which facial muscles are relaxed [30] or when the face is not explicitly and voluntarily expressing a mental state. Though these expressions are suggested to express no emotion, it has been documented that they can trigger physiological responses among individuals (e.g., [31]) and/or may be interpreted as emotional, particularly by anxious individuals [32]. This might be due to the ambiguity of such an expression. Neutral expressions are indeed uncommon in the sense that we are rarely confronted to the absence of expression on faces. Another evidence that face with no expression is odd stems from studies that investigated the “still face” [33], which showed that during a face to face interaction between a mother and her child, the child displayed distress behaviours (e.g., crying) when the mother did not express anything.

## Methods

In order to improve the searching process, the review used the PRISMA Checklist [34] as guidelines for a systematic and comprehensive review of alexithymia and EFE processing (see PRISMA Checklist S1 and PRISMA Flow Diagram S1).

### Literature search

Only studies that were indexed on Psycinfo, PubMed, and Web of Science, from 1990 to 2010 were selected. We used a combination of keywords: “*perception, recognition, identification, differentiation, detection, discrimination, decoding, faces, emotional facial expressions, alexithymia*”.

### Selection criteria

We selected only studies that were published in English in international peer reviewed journals. We only included studies that measured alexithymia using one of the most widely used self-report questionnaires (TAS-26 and TAS-20 [35]; BVAQ [36] and BVAQ-B), and that did not use EFEs as primes in interference/facilitation tasks. The validity of the TAS-26 has been questioned: one factor is correlated with social desirability and the validity of another factor is not supported by factorial structure analyses [35]. Thus, the studies that used the TAS-26 should be interpreted with caution, relative to other studies that used the TAS-20 and the BVAQ. Relative to the TAS-20, the BVAQ includes two additional factors named fantasizing and emotionalizing. Fantasizing attempts to directly assess the facet involving imagination processes and the emotionalizing assesses the degree to which a person can be emotionally aroused by emotion-inducing events [36].

### Data Extraction and study characteristics

Relevant data were independently extracted by one of the authors and checked by a second for accuracy. The following variables were extracted and are presented in Table 1: (1) the type of task (labelling, matching, signal detection, gender categorization, masked presentation, or passive viewing- these terms will be defined later), (2) the database from which the EFEs were taken, (3) the type of discrete EFEs, (4) the number of EFEs presented for each emotion, (5) the nature of stimuli presentation (static pictures or dynamic videos), (6) the duration of presentation, (7) the population (clinical or healthy), (8) the sample size, (9) the alexithymia scale used, (10) the statistical approach used (categorical or dimensional), (11) potential confounding affective dimensions that were controlled for, (12) the behavioural results, (13) the neuroimaging statistical analysis (whole brain; regions of interest, ROI), and (14) the neuroimaging results.

## Results

### Included studies

The PRISMA Flow Diagram S1 shows the selection process. Using the above mentioned keywords, forty one studies were listed, out of which thirteen did not fulfil the inclusion criteria: eleven did not measure alexithymia or did not provide information about how alexithymia was associated with performance and/or brain activity [37], [38], [39], [40], [41], [42], [43], [44], [45], [46], [47], and two used the Schalling-Sifnéos Personality Scale to measure alexithymia [48], [49], the validity of which has been questioned [20]. Finally, four studies measured behaviours other than the decoding of EFEs [50], [51], [52], [53]. Consequently, twenty-four studies satisfied all our inclusion criteria. None of the studies reported effect sizes, preventing the examination of this issue in the present review.

### Summary of findings

#### Clinical populations

All the studies we found used explicit tasks, i.e. which require participants to consciously and deliberately process and/or respond to the emotional aspect of the EFE. These studies all referred, more precisely, to labelling tasks, that require participants to select among several emotional labels the one that best fits a particular expression. Within this section, we will always first give group differences in terms of alexithymia level and performance (main effects), and then report the associations between alexithymia scores and performances.

Mann, Wise, Trinidad, and Kohanski [54] compared substance abusers (of alcohol and/or other substances) and controls. Substance abusers showed higher levels of alexithymia, but did not differ from the control group in terms of labelling EFEs. Moreover, alexithymia was not related to the labelling of EFEs. Galderisi et al. [55] showed that patients with panic disorder had higher DIF and DDF scores than healthy controls. However, the two groups did not differ in their ability to decode ambiguous schematic faces (i.e., faces that displayed similar proportions of positive and negative emotions), and alexithymia did not predict decoding performance ([55], personal communication).

In these studies, there were no differences between healthy and clinical groups in terms of performances. Moreover, even though groups differed in terms of alexithymia, alexithymia was not linked to labelling abilities.

Kessler et al. [56] and Pedrosa Gil et al. [57] used the Facially Expressed Emotion Labelling paradigm (FEEL [58]) in which one EFE is presented right after the presentation of a neutral face of the same person. Participants have to label the target EFE, with higher score indicating better performance. Kessler et al. [56] reported higher levels of alexithymia among eating disorder patients than healthy controls (except for the EOT subscore). However, these two groups had similar FEEL performances and the authors failed to find any association between alexithymia scores and the FEEL performances in either group. Pedrosa Gil et al. [57] reported greater difficulties in identifying feelings (DIF subscore) and worse FEEL performances among somatoform disorder patients relative to healthy controls. Yet, when TAS-20 total scores were entered as a covariate, the group effect for FEEL performances disappeared. This suggests that alexithymia explains the performance differences between somatoform disorder patients and healthy controls. Moreover, pooling the two samples together, there was a negative association between the TAS-20 total scores and the total FEEL scores.

Kätsyri, Saalasti, Tiippana, von Wendt, and Sams [59] assessed the processing of EFEs in adults with Asperger syndrome and healthy controls by varying the perceptual quality of EFEs. The perceptual quality of EFEs was based on manipulations of spatial frequency: normal, slight filtering, and strong filtering. Slight and strong filtered images are respectively slightly or strongly low-pass filtered (which results in global shapes without local features). The EFEs used were either static or dynamic. Compared to controls, the group with Asperger syndrome had higher alexithymia scores (TAS-20 total, DIF, DDF and EOT). They were also less accurate in labelling strongly degraded EFEs. However, this latter group effect was no longer significant when alexithymia scores were entered as a covariate (Interaction between group and degradation: *F*(2, 74) = 0.04, *p* = .96); effect of group on strongly degraded images: *F*(1, 37) = 0.38, *p* = 0.54; effect of group on the index of degradation: *F*(1, 37) = 0.01, *p* = .93; [59], personal communication). This result suggests that alexithymia accounts for the deficit that people with Asperger syndrome have in labelling EFEs. Moreover, among the whole sample, alexithymia scores (TAS-20 total and DIF, DDF and EOT) were found to be positively correlated with the index of degradation, meaning that high alexithymia scorers are particularly impaired when EFEs are degraded relative to non degraded EFEs.

Ridout et al. [60] assessed the association between alexithymia and emotion labelling abilities among non clinical participants with high versus low vulnerability for disordered eating (i.e., self-reported scores on the Eating Disorder Inventory, which measures drive for thinness, bulimia, and body dissatisfaction). They used The Awareness of Social Inference Test (TASIT [61]), which includes an emotional labelling task. Participants had to label the emotion (the six basic emotions and neutral expressions) displayed in videos. Results showed that participants with subclinical disordered eating had higher alexithymia scores and lower labelling skills (total scores for all expressions and anger) than the other group. In the entire sample, there was a negative correlation between alexithymia (TAS-20 total scores) and the ability to label angry expressions. Moreover, in a regression analysis with drive for thinness, bulimia, body dissatisfaction, TAS-20 total and anxiety scores as predictors of labelling performances, only the TAS-20 total score and severity of bulimia scores were significant negative predictors of global labelling abilities (total score for all EFEs), whereas only body dissatisfaction predicted poor anger labelling. In addition to the level of disordered eating symptomatology, alexithymia severity is thus a relevant predictor for the global labelling of EFEs but not for anger labelling in disordered eating.

At the cerebral level, Pollatos, Herbert, Schandry, and Gramann [62] examined visual-evoked potentials (VEP) to emotional faces during a labelling task in patients with anorexia nervosa and controls. VEP are a subclass of Event-Related Potentials and refer to brain responses to visual events (in this study, EFEs). The authors investigated the P300 and the N200, which index respectively the perceptual processing and the allocation of attentional resources [63]. Relative to controls, the patients showed higher levels of alexithymia (as measured by TAS-20 total, DIF and DDF scores). With respect to labelling EFEs, patients were less accurate than controls when alexithymia and depression scores were modeled in the analyses. Moreover, in the entire sample, DIF and DDF scores were negatively correlated with the ability to process neutral faces. With respect to the VEP indices, there was no association between any alexithymia factors' scores and the amplitude or latency for any event-related potentials.

The above studies demonstrate that a variety of disorders are marked by high levels of alexithymia. All reviewed studies showed differences in terms of alexithymia but some of these studies did not show group differences in terms of labelling. However, when there was an effect of the clinical diagnosis on the labelling scores (Asperger syndrome, eating disorders, and somatoform disorders), alexithymia always predicted difficulties in labelling EFEs. These difficulties apply to both static and dynamic EFEs, and to EFEs that are characterized by poor or high signal quality (slight and great amount of perceptual information). Moreover, the deficits may extend to neutral facial expressions. In order to clarify these issues, we examined studies that focused on the association between alexithymia and EFEs processing in healthy individuals, at both the behavioural and cerebral levels.

#### Healthy populations: Behavioural studies

The behavioural studies conducted with healthy populations all used explicit tasks, including labelling, matching, and detection procedures. Matching task (when it includes only EFEs) refers to the identification of a previously presented target face among distractors. It relies more on visuoperceptual comparisons than semantic understanding of emotional information [64], and as such, it involves less semantic processing and more memory processing than labelling tasks. Detection tasks involve the selective identification of an emotional expression among other sequentially presented expressions (emotional or neutral) and relies mainly on visuoperceptual abilities.

Relative to the labelling tasks, we found eight studies in which participants were instructed to label the emotion. Five of them used the Pictures of Facial Affect (PFA) data base [30].

Out of these five studies, only that of Prkachin, Casey, and Prkachin [65], who used a dimensional approach, failed to find any correlation between alexithymia scores and EFE labelling performances. Mann, Wise, Trinidad, and Kohanski [66] compared high vs. low alexithymia scorers (HA vs. LA, respectively) and showed that HA were impaired in labelling EFEs, particularly for sadness. Jessimer and Markham [67] also found that HA had lower labelling performances than LA. Montebarocci, Surcinelli, Rossi, and Baldaro [68] were able to demonstrate that HA were worse at labelling EFEs, even after controlling for the fact the HA also had greater depression and anxiety scores. However, this effect did not remain after controlling for verbal abilities. This is the only study which controlled for verbal abilities.

Swart et al. [69] tested whether people with alexithymia could improve their labelling of micro facial expressions with a training paradigm known as the “Micro Expression Training Tool”. In the first test phase of this paradigm, two neutral facial pictures are simultaneously presented. Then, both pictures gradually evolve to a specific emotional expression (e.g., anger and disgust). The paradigm consists of presenting pairs of facial expressions that are commonly confused with each other (anger/disgust, contempt/happy, fear/surprise, fear/sadness). This phase aims to train participants to notice the differences between the expressions that are often confused. In the second test phase, the display involves a single neutral face that rapidly (15 msec) evolves to a specific emotional expression before returning to the original neutral expression. Participants have to label the EFE and they receive feedback on their performance. Pooling all the EFEs together, these authors reported that, despite training, individuals who have been selected for their high levels of DDF (from the BVAQ) were less accurate than those with no such difficulties. However, there were no baseline measures of EFE processing before training, leaving us unable to conclude whether the training may have nevertheless improved the processing of EFEs in alexithymia.

Using an unstandardized EFE database and a comparison of two small groups of participants, Pandey and Mandal [70] found no differences in labelling performance between HA and LA.

Relative to matching tasks, Lane et al. [71] created the Perception of Affect Task (PAT) and investigated whether alexithymia is associated with difficulties in matching combinations of verbal and non-verbal emotional material (labels and vignettes as well as EFEs and emotional scenes). This matching task was thus not limited to EFEs. Results indicated a negative association between TAS-20 total scores and emotional processing on each subtask, which was not affected by anxiety and depression levels. These results were among the first to confirm that alexithymia is associated with a general impairment in processing emotional information and is not limited to labelling impairment. Yet, the question of whether some emotions might be particularly difficult to process was not addressed in this study.

In another set of analyses, Lane et al. [72] examined if there was a specific effect of the emotional category (irrespective of the subtask). They showed that alexithymia was associated with poorer performance on the PAT for all the six emotions (anger, fear, disgust, sadness, happiness, and surprise), as well as for the neutral material. However, the fact that these authors did not separately analyze the subtasks involving EFE precludes making strong conclusions about deficits in matching specific EFEs, with scenes, vignettes or labels.

Relative to signal detection and matching task,. Prkachin et al. [65] further investigated the links between alexithymia and EFE processing with a task that mixed signal detection and matching instructions. On each trial, one of the six basic EFEs (target) was presented, followed by six different EFEs of different people, which were presented successively for 33 milliseconds each. Participants indicated whether one of the six non-target faces displayed the same expression as the target face. A score of perceptual sensitivity was calculated by taking into account the rate of hits and false alarms for the detection of each of these emotions. Higher sensitivity scores indicate more accurate detection. Results showed that TAS-20 total and EOT scores were negatively correlated with sensitivity scores for each of the six EFEs. High DDF scores were linked to worse performance in matching sadness only.

Relative to signal detection task, Parker, Prkachin, and Prkachin [73] conducted another study using a perceptual sensitivity score. In this study, participants viewed pictures of sad, angry, fearful or neutral faces that they looked at for either 1 or 3 seconds. The authors found that increasing levels of DDF were associated with decreasing sensitivity scores for negative, but not neutral faces, in the 1 second condition only. Importantly, this association was observed though partialling out the effect of positive and negative affectivity.

In sum, alexithymia is associated with deficits in detecting and matching EFEs presented briefly. Alexithymia is also associated with impairments in matching emotional and neutral facial expressions, labels, scenes and vignettes. Relative to the labelling tasks, not all studies supported deficits among healthy HA. Moreover, verbal abilities may account for labelling deficits.

#### Healthy populations: Neuroimaging studies

The majority of neuroimaging studies investigating alexithymia and EFE processing involve instructions that do not explicitly require the processing of the emotion displayed on the face. That is, participants were instructed to either make no decision or make a decision on emotionally-unrelated perceptual or categorical features of stimuli that varied in emotional content (e.g., judging the gender of the face). The presentation of the following neuroimaging studies is organized according to the specific procedure employed in each study: masked perceptual processing, gender categorization, passive viewing and categorical perception. Each procedure will be explained in its corresponding subsection.

Masked perceptual processing involves a very short presentation of an EFE (prime), which is not supposed to be consciously perceived by participants, followed by a longer presentation of a neutral expression (target). Out of the four studies we found [74], [75], [76], [77], three [74], [75], [76] used the same masking paradigm, where neutral target faces were primed for 33 milliseconds by either a sad, happy or neutral face, or no face at all. Participants had to rate the neutral mask faces as expressing negative or positive feelings. None of these three studies found an effect of alexithymia or prime on these ratings.

Eichmann et al. [74] focused on the fusiform gyrus as a region of interest, an area that has been consistently implicated in the processing of emotional and non emotional faces (e.g., [78], [79]). They found a negative correlation between the TAS-20 total and DIF scores and the fusiform gyrus' level of activity for sad faces. They did not find any effect with happy faces.

Kügel et al. [75] focused on the amygdala as a region of interest, which is a key structure for the processing of emotional stimuli - notably EFEs (e.g., [80]). There was no association for the happy faces, but there was a negative correlation between the TAS-20 total and DIF scores and the amygdala's level of activity (bilaterally) for sad faces. The same association was reported for the DDF scores, but only with the left amygdala. When depression and anxiety scores were entered as covariates, only the association between DIF scores and right amygdala activation remained significant.

Reker et al. used the same experimental design, but they enlarged their sample size and conducted whole brain analyses [76]. For sad faces, they found a negative association between the level of alexithymia (using TAS-20 total, DIF and DDF scores) and the activity in both the amygdala (left) and the fusiform gyrus response after partialling out the effect of anxiety and depression. Moreover, other negative associations were found between the TAS-20 total scores and the response of regions involved in spatial processing (e.g., parahippocampal gyrus - BA37), emotion identification of visual stimuli (middle occipital gyrus - BA19 [81], [82]), in the extraction of the meaning of EFEs [83], as well as in regions that have been involved in the understanding of another's mental state [84], [85] (e.g., insula; superior temporal gyrus - BA41, inferior frontal gyrus - BA47).

During the processing of happy faces, Reker et al. [76] found similar associations between the total alexithymia score and brain activation than during the processing of sad faces (i.e., insula, inferior frontal gyrus, superior temporal gyrus, parahippocampal gyrus, and middle occipital gyrus). In addition, they found a negative correlation between alexithymia (TAS-20 total scores) and activation in regions involved in somatosensorial integration (claustrum, superior parietal lobule - BA7 [86], [87]). Importantly, for both sad and happy faces, the effects remained significant when controlling for anxiety and depression scores.

Duan, Dai, Gong, and Chen [77] used the same masking paradigm than the above mentioned studies, but with EFEs of surprise (an emotion that had not been investigated previously), happiness, and neutral. Unlike Reker et al., but consistently with Eichman et al. and Kügel et al's studies, there was no association between alexithymia scores and the neural responses to happy faces. For surprise, the results resembled those previously reported for sadness, with a negative association between the level of alexithymia (DIF scores) and activity in regions involved in spatial processing (parahippocampal gyrus), in faces processing (e.g., fusiform gyrus) and more specifically in the processing of EFEs (superior temporal gyrus).

To sum up, these studies that used the masking procedure all showed that during the processing of negative EFEs, alexithymia, and in particular the DIF and DDF factors, were associated with lower activation in brain regions involved in spatial attention, in the emotional identification of visual stimuli, in the processing of faces (emotional or not) and in understanding another's state of mind or action. Regarding positive EFE, similar associations than with sad faces have been reported for surprise (but this was investigated in only one study), but for happy faces, the results are inconsistent and only one study (out of four) suggests HA is associated with less responses to happy faces in regions involved in sensory integration.

Gender categorization tasks require participants to judge the gender of the person who expresses a neutral or an emotional facial expression. In the study of Kano et al. [88], participants were scanned while viewing faces that varied in emotional intensity (i.e., 33%, 67%, and 100%). After each scanning session, participants were asked to evaluate each target face in terms of its intensity of anger, sadness, happiness, disgust, fear or surprise. Analyses of the behavioural data showed that HA were more likely to rate the most intense sad faces (100%) as showing disgust, but this was the only effect that differed from that of the LA group. At the neural level, we will only present the analyses that used a contrast with neutral expressions as the main purpose of this present review was to investigate the links between alexithymia and the processing of EFEs. Though, all activations are presented in Table 1. The results showed that the contrast between angry (regardless of their intensity) and neutral expressions induced less activity in HA (vs LA) in the superior temporal gyrus (BA42), the middle occipital gyrus (BA19), the insula, the cerebellum, the precentral gyrus (BA4) as well as in the dorsal anterior cingulate cortex (BA32). Relative to neutral faces, sad and happy faces (regardless of their intensity) induced less activity in the insula in HA (vs LA). HA (vs LA) also had less activation in the medial frontal gyrus (BA10) in response to happy faces. Therefore, this study suggests that alexithymia is associated with weaker responses in regions normally activated during the processing of EFEs (insula and superior temporal gyrus) and during representation of one's own emotional responses (precentral gyrus; medial frontal gyrus; [89]). Furthermore, alexithymia was associated with lower activity in the dACC, a region which play a role in conflict processing in an emotional context [90], [91]. Thus, if we conceive that processing the emotional expression is not voluntary, lower activation in this region among HA might suggest that alexithymia is associated with reduced conflict between the emotional expression that is task irrelevant and the gender (task relevant).

Mériau et al. [92] examined the association between alexithymia and brain activation to angry and fearful faces during two experimental conditions: while doing a gender vs an emotional categorization task. For both tasks, they found no association between alexithymia and the behavioural measures (reaction times and correct responses). At the neural level, during the gender categorization task and pooling across the fear and anger stimuli, the alexithymia total score was positively correlated with activity in the dACC (BA8 and BA32). This effect remained significant even after controlling for anxiety and positive and negative affectivity scores. No covariation of alexithymia total score with brain activity was found during emotion decisions. Further, these authors split the sample into a LA and HA group (median split) to investigate if alexithymia modulates task-dependent changes in effective connectivity with dACC. In LA, the coupling of dACC with ventro-lateral prefrontal cortex was greater during both emotion and gender tasks, and with dorso-lateral PFC only during emotion categorization. According to the authors, this pattern of connectivity indicated that LA had a more “*elaborated evaluation of EFEs*” ([92] p. 1025). Conversely, HA showed greater coupling of dACC with the amygdala during both tasks, which has been interpreted by the authors as an ‘*increased affective influence on the dACC to enhance information processing by guiding attention to salient emotional cues*’ ([92] p. 1025).

The observed pattern of dACC/AMG connectivity among HA, together with the reported positive association between the level of alexithymia and the level of dACC activity during gender categorization, could reflect greater orientation to the emotional cues among the HA. However, this hypothesis should be further tested as the results of Mériau et al. [92] contradict those of Kano et al. [88] who showed that alexithymia was negatively associated with dACC activation. The discrepancy between these two studies may result from several methodological differences such as the instructions (assess the intensity of EFEs after each block in [88] vs no emotional intensity assessment in [92], the database of EFEs : ATP in [88] vs PFA in [92]), the emotions displayed (sadness, anger, and happiness in [88] vs anger and fear in [92]).

During a passive viewing task, participants are instructed to passively look at the stimuli but not to judge them. In Heinzel et al. [93] study, participants were presented with fearful, happy and neutral facial expressions, and were instructed to just press a button when they had “*recognized the picture*”. These authors showed no behavioral differences between HA and LA, but showed that, HA (relative to LA) had increased activation in the dACC in response to both fear and happy faces. Hence, it is possible that HA might have tried to “*recognize the picture*” in terms of gender rather than in terms of emotion but may still have oriented their attention to the emotional cues. This hypothesis is also in accordance with the findings and suggestions of Mériau et al. [92].

Categorical perception is defined as the ease of discriminating two stimuli from two different categories (between-category pairs) relative to discriminating two stimuli belonging to the same category (within-category pairs) [94]. Vermeulen et al. [95] assessed whether alexithymia was linked to an attentional delay to detect an infrequent EFE among frequently presented EFEs. These frequent EFEs displayed 65% of expression A and 35% of expression B. Sometimes the infrequent expressions shared the main emotional expression as the frequent one (within condition; 95% of expression A and 5% of expression B), and sometimes they do not (between condition 35% of expression A and 65% of expression B). The authors used morphed disgusted and angry faces in an oddball paradigm (infrequent EFE detection) and collected VEP indices, in particular the N2b and P3a components. The N2b component is believed to reflect an attentional orientation to encode new information. The P3a component is associated with detecting the degree of perceptual deviation of a novel stimulus relative to frequent stimuli [96].

Vermeulen et al. observed that HA had longer N2b component latencies than LA, for both disgust and anger, even after controlling for depression and positive and negative affectivity scores. Moreover, unlike the LA, the HA displayed similar N2b latencies for between and within conditions with anger. Finally, the P3a component, which is sensitive to changes in the deviation of a novel stimulus, was delayed in HA for both disgust and anger. These results suggest that HA had difficulties in detecting change and novelty in angry expressions.

## Discussion

### Objective 1 and 2: are alexithymia or clinical disorders better predictors of EFE decoding deficits?

In the present review, out of the seven studies conducted in clinical populations, four showed that alexithymia was associated with deficits in the labelling of EFEs, the same four studies that found lower scores for labelling EFEs in clinical populations than in controls. Importantly, three of these studies have attempted to disentangle the effects of depression, anxiety and alexithymia. These studies revealed that alexithymia partially or even totally explained the between-group differences in the labelling of EFEs (alexithymia as a covariate or as a predictor in regression analyses) [57], [59], [60]. This suggests that the emotional recognition impairments that have been reported in a variety of clinical disorders may be partly attributed to comorbid alexithymia rather than to the clinical status of the individual, particularly for Autism Spectrum and Eating Disorders.

Although alexithymia may account for some of the relationship between clinical disorders and emotional labelling deficits, it may also be the case that clinical disorders can account for some of the relationship between alexithymia and emotional labelling deficits. One study that tested this latter possibility was conducted by Ridout et al. [60], who demonstrated that when the level of anxiety and the severity of eating-disorder symptoms were modelled, alexithymia remained a significant predictor of global EFE labelling performance, although it was no longer a significant predictor of anger labelling (which appears to be more closely related to body dissatisfaction). Thus, alexithymia significantly predicted labelling impairments of EFEs in general, even if other personality constructs may predict impairments for specific EFEs. These results illustrate that in order to ascertain whether alexithymia accounts for some patients' inability to understand others' emotions, it is important to also estimate the impact of other comorbid psychological symptoms. We believe this is notably the case for anxiety and depression disorders, as the clinical populations that have been investigated with regards to emotional processing generally not only exhibit high levels of alexithymia (e.g., [97], [98], [99]), but also of depression and anxiety (e.g., [100], [101], [102]). Moreover, non-clinical HA are prone to report greater levels of depression and anxiety (e.g., [103], [104]), which are themselves associated with EFE processing deficits (e.g., [28], [105]).

In the present review of the studies conducted with healthy participants, several studies have measured dysphoric affects as well as positive and negative affectivity to eliminate these primary sources of confounds in their research. All these studies demonstrated that alexithymia is indeed linked to specific behavioural or neural responses during EFE processing, after controlling for the above confounding dimensions. Taken together, the results of these studies support the idea that mood disorders and alexithymia share common variance, but are dissimilar constructs.

In addition to depression and anxiety disorders, social anxiety may also account for the relationship between alexithymia and EFE processing impairments in particular in Autism Spectrum and Eating Disorders. Both types of patients present frequent comorbid social anxiety disorders (e.g., [106], [107], [108], [109]) and alexithymia has been related to social anxiety (e.g., [110], [111]). Social anxiety has been associated with biases in the interpretation of facial expressions (e.g., [32], [112], [113]). This could account for the alexithymia deficit to correctly label neutral EFEs, as demonstrated in one third of the studies that included a neutral face condition [62], [72]. Thus, disentangling the influence of social anxiety from that of alexithymia might be a critical issue in future research on alexithymia and EFEs processing, notably in Autistic Spectrum and Eating Disorders. However, the association between alexithymia and neutral faces processing has to be interpreted with caution. In fact, most of the above mentioned studies used significantly more emotional than neutral faces. This may induce a response bias, i.e. lead the subjects to use an emotional label rather than a neutral one when decoding EFEs. Therefore, lower performances in identifying neutral expression might result from a tendency to select an emotional label rather than a true deficit in decoding neutral expressions.

In sum, regarding our first and second objectives, this review provides empirical support for the idea that alexithymia is linked to emotional expressions labelling deficits independently of the clinical condition of the participants and of comorbid dysphoric affects.

### Objective 3: Level of processing

Another objective of the present review was to determine which level(s) of EFE processing (e.g., perceptual abilities; representation of emotion concepts) may be affected in alexithymia and in thus in which type of tasks these deficits appear. Whereas the studies in clinical groups involved mainly explicit tasks (processing of emotional information on EFEs), studies among healthy participants with high alexithymia scores used a wider range of tasks (processing of emotional vs. non-emotional information on EFEs) and processes (perceptual abilities; representation of emotional concepts).

#### Processing of emotional information on EFEs

All methodological heterogeneity considered, most of the studies demonstrated a consistent relationship between alexithymia and the ability to decode others' emotions: that is, HA is associated with impairments in detecting, matching and labelling EFEs. As mentioned previously, matching and detecting rely mainly on a perceptual analysis of EFEs. Labelling tasks involve perceptual analysis as well, but they also rely on the semantic understanding of emotional information. Because of the overlap in the processes involved in matching, detecting and labelling, the deficits in these tasks will be described in terms of the processes that are implicated.

Relative to the perceptual properties of EFEs, the present review highlights that alexithymia is associated with poorer EFE processing if facial expressions are displayed under suboptimal conditions, i.e. when the stimuli were spatially degraded [59] or briefly presented above the threshold of conscious detection [65], [73]. These results suggest that HA may need more information [59] and/or more time to correctly identify the EFE [65], [73]. Kätsiry et al. [59] showed that alexithymia was associated with impaired identification of degraded EFEs. They did not find any significant correlations with non-degraded EFEs. This may mean that the perceptual cues that define emotional expressions are not well represented among HA. Weaker representations of EFEs may also account for why alexithymia is associated with deficits in detecting and labelling EFEs presented for durations of 1 second or less. It may be that HA need more time to correctly detect or label EFEs because they are less interested in these expressions, and thus less familiar with them.

The time required by HA to process EFE may also be critical if HA use different strategies to discriminate EFEs, compared with LA [95]. For example, Bird, Press, and Richardson [114] recently investigated the potential impact of alexithymia on joint attention deficits in individuals with an autism spectrum disorder. In this study, participants' eye fixations were measured as they viewed short videos excerpts that depicted social interactions. Individuals' alexithymia scores were negatively correlated with the number of fixations to the eye region (relative to the mouth region), but their pattern of attention allocation was not related to the severity of their autistic symptoms. Allocating less attention to the eye region may compromise the ability of HA to process EFEs because expressions conveyed by the eyes are core features for the communication of mental and emotional states [115], [116]. For example, it has been demonstrated that the eye region is the first facial area to be processed during an emotional categorization task [117], suggesting that the eyes convey important information used to identify emotions. Furthermore, focusing on eye gaze affects people's perception of emotional intensity (see [118]), which could account for why HA tend to show reduced fear intensity ratings [65].

Another argument that the allocation of attentional resources to EFEs is modulated by alexithymia stems from a study by Vermeulen et al. [95] using (VEP). They showed that HA exhibited normal behavioural performance (correct identification of infrequent facial expressions among frequent facial expressions; no response time differences between groups for the identification of expressions). We might suggest that there was no behavioural evidence of attentional difficulty because the conditions were not constrained enough (1 error on 256 deviant trials). However, the VEP analyses showed that HA had similar N2b latencies for between and within conditions with anger and showed a delayed P3a for both disgust and anger. These results support the hypothesis that alexithymia is associated with difficulties in detecting perceptual differences, at least within the expression of anger. We may speculate that the delayed sensitivity to the degree of novelty of deviant stimuli in HA results from fewer attentional resources being allocated to emotional changes and their associated facial areas, such as the eye region. This difficulty in identifying perceptual differences between different EFEs is also thought to be present in individuals with Autism Spectrum condition, as they have problems in recognizing the “*internal affective state of another individual, independent of their identity, via the shape of individual features and changes in their relative distance from one another during the expression*” ([119], p.128).

Thus, it could be that HA may not be impaired in detecting, matching, or even labelling EFEs *per se*. Instead, HA may have deficits in processing the perceptual properties of EFEs. These deficits may be evident only when the quality of the signal is poor and when HA do not orient their attention to the eye region.

Relative to semantic understanding, Lane et al. [71] found that high alexithymia scorers have difficulties in extracting information from different types of stimuli (verbal and non verbal) that are not necessarily emotional. They found that HA were worse than LA in matching emotional (and neutral) stimuli with emotional (and neutral) responses. For example, participants had to match an EFE with a scene that depicts an emotional situation without containing EFEs. These findings raised the question of whether alexithymia is associated with a general deficit in extracting the emotional (and non emotional) nature of vignettes or visual scenes, rather just being specific to the processing of EFEs. Thus, it may be that alexithymia is associated with weaker representations of both emotional and non-emotional stimuli. One way to test this hypothesis may be to examine whether abstract verbal reasoning and representations of verbal concepts vary accordingly with alexithymia levels.

The present review raises the question of whether verbal abilities may constitute a source of confounds of the effect of alexithymia on labelling performances. Montebarocci et al. [68] showed that the relationship between alexithymia and labelling ability in healthy individuals was mediated by verbal ability (verbal IQ). In line with the literal meaning of the word ‘alexithymia’ (Grec; *a* = lack, *lexis* = word, *thymos* = mood), there is converging evidence showing that HA use fewer words to describe their emotional experiences than LA (e.g., [120], [121]). With respect to the literature on the associations between alexithymia and verbal IQ, results have been contradictory: some studies found an association (e.g., [122], [123]), but others did not (e.g., [124], [125], [126]). These discrepancies could be due to the fact that different studies used different questionnaires to measure alexithymia (e.g., TAS-20: [122], [123]; TAS-26: [124]; BVAQ: [126]). Nevertheless, at least in some psychiatric disorders such as Autism, the question of whether low IQ affects EFE processing may be important. In fact, in the study of Kätsyri et al. [59], lower verbal IQ scores in the group of patients with Asperger Syndrome could have accounted for the impaired ability of HA to label EFEs instead of alexithymia. Hence, taken together, the above mentioned studies suggest that HA may have impaired representations of emotional concepts (and perhaps of neutral concepts as well), which suggests that the impact of verbal ability should be controlled for in future research.

#### Processing of non emotional information on EFEs

Masking studies consist in presenting rapidly an EFEs which is then masked by a neutral expression so that participants are not aware of the EFE. These studies demonstrated a negative association between the level of alexithymia and the response of core brain regions involved in the processing of EFEs, particularly those involved in the processing of faces that are emotional or not (e.g., amygdala, fusiform gyrus, insula, the superior temporal gyrus, the occipital cortex). Masking studies allow the assessment of “ (…) *the automatic encoding of emotional information and/or the generation of (…) emotional responses*” ([76], p.2). Thus, lower activation in regions involved in the processing of visual emotional stimuli revealed that HA have weaker perceptual representations of EFEs [76]. This result is in accordance with other behavioural studies [59], [65] which highlighted perceptual impairments among HA during the processing of EFEs.

More specifically, the present review demonstrates that HA exhibit lower activation in both the fusiform gyrus and the amygdala, two structures that have been shown to play a critical role in the early stage processing of facial expressions (see [127] for a review). More critically, there is increasing evidence, from both functional and morphological neuroimaging studies, that the combined action of these two structures underlies efficient EFE processing (e.g., [128]). In his model on the physiopathological mechanisms that may influence the emergence of the social deficits that are characteristic of Autism Spectrum condition, Schultz [119] argues that the fusiform-amygdala system is a cornerstone for the development of competent social cognition and harmonious interpersonal relationships, and that this system is deficient in autism. In line with this model, and on the basis of more recent data suggesting that awareness of an emotional stimulus is associated with concerted activation of these two structures [129], Dziobek et al. stated that “*altered connectivity between the amygdala and fusiform gyrus, therefore, may not only lead to the impairments in face recognition but also to impairments in emotional awareness as indicated, for example, by higher levels of alexithymia in individuals with autism spectrum conditions*” ([128], p. 403). Thus, investigating whether abnormalities in the fusiform-amygdala system could be responsible for the specific response patterns of these other structures may be a promising area for future research. These studies also showed lower activation in HA (vs LA) in regions involved in the sensory integration and in the human mirror neuron system (e.g., inferior frontal gyrus). Lower activity in the inferior frontal gyrus suggests that HA do not generate in themselves the emotion displayed (i.e., experience the emotion) by the face when the instruction does not specify an explicit processing of EFEs.

In contrast to the masked studies, which have demonstrated a consistent relationship between alexithymia and brain areas implicated in the processing of EFEs, gender categorization studies have shown contradictory findings. For example, Kano et al. [88] showed lower activation in regions involved in the processing of faces in general (superior temporal gyrus; BA42; [130]), of EFEs (middle occipital gyrus, BA19, [131]) and more specifically of disgust expressions (insula; [132]) in HA (vs LA) during a gender decision task. Kano et al. [88] also found activation in regions involved in threat detection (e.g., cerebellum, [133]), in interoceptive awareness (precentral gyrus, BA4; [134]). They also showed that HA have reduced activity in the dorsal ACC, suggesting that they have less interference from emotional expressions. In contrast, Mériau et al. [92] showed that HA exhibit greater activity in the same region when performing the same type of task. These contradictory findings do not allow us to conclude whether HA exhibit greater interference from emotional cues during a gender classification task.

In sum, the masked studies show that alexithymia is associated with lower activation in regions generally activated during task-irrelevant processing of EFEs. However, other studies are needed in order to test the hypothesis of greater (or weaker) interference of task-irrelevant emotional cues among HA on the processing of task-relevant neutral cues.

### Objective 4: Discrete emotion

**Figure 1:**
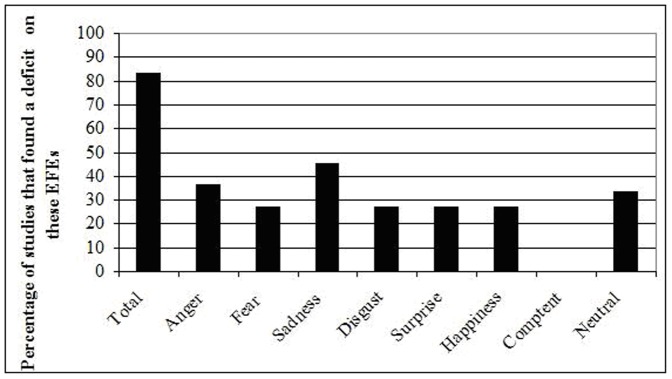
Behavioral deficits (in percentage) among high alexithymia scorers to process emotional and neutral facial expressions, without controlling for confounding factors. The deficits refer to the number of studies that found a deficit for the emotion relative to the number of studies that assessed this emotion.

The final objective of the present review was to determine whether the deficit in processing EFEs in alexithymia applies to specific EFEs, or rather to all EFEs, including neutral ones. As illustrated in Table 2 and Figure 1, it appears that impairments in processing EFEs in alexithymia are global, i.e., that the deficits concern not only negative emotions [60], [65], [66], [67], [72], [73], but also happiness [65], [67], [72] and surprise [65], [67], [72]. This global deficit is also reflected in the neural responses of HA, who show less activation in brain areas associated with the processing of facial expressions for negative emotions [74], [75], [76], [88], [92], [93], [95] but also happiness [76] and surprise [77]. An inspection of the proportion of studies that observed a deficit in processing EFEs highlights a gradient across the negative EFEs: 45.5% for sadness, 36.4% for anger, 27.3% for fear and 27.3% for disgust. Yet, based on these results, it would be premature to conclude that alexithymia is associated with a deficit in processing a particular emotion and additional studies should clarify this issue, for instance, by conducting a meta-analysis.

**Table 2:** Behavioural deficits among High Alexithymia Scorers to process emotional and neutral facial expressions, without controlling for confounding factors.

	Total	Anger	Fear	Sadness	Disgust	Surprise	Happiness	Contempt	Neutral
**1. Mann et al. ** [**66**]	–	x	x	–	X	x	x	n.i.	n.i.
**2. Mann et al. ** [**54**]	x	x	x	–	X	x	x	n.i.	n.i.
**3. Lane et al. ** [**71**]	–	Ø	Ø	Ø	Ø	Ø	Ø	n.i.	Ø
**4. Pandey and Mandal ** [**70**]	n.i.	x	x	x	X	x	x	n.i.	n.i.
**5. Jessimer and Markham ** [**67**]	–	–	–	–	–	–	–	n.i.	n.i.
**6. Lane et al. ** [**72**]	–	–	–	–	–	–	–	n.i.	–
**7. Parker et al. ** [**73**]	–	Ø	Ø	Ø	n.i.	n.i.	n.i.	n.i.	n.i.
**8. Kessler et al. ** [**56**]	x	x	x	x	X	x	x	n.i.	X
**9. Pollatos et al. ** [**62**]	n.i.	x	x	x	X	x	x	n.i.	–
**10. Prkachin et al. (study 1) ** [**65**]	n.i.	–	–	–	–	–	–	n.i.	n.i.
**11. Kätsyri et al. ** [**59**]	–	Ø	Ø	Ø	Ø	Ø	Ø	n.i.	n.i.
**12. Pedrosa Gil et al. ** [**57**]	–	x	x	x	X	x	x	n.i.	n.i.
**13. Ridout et al. ** [**60**]	–	–	x	x	X	x	x	n.i.	X
**14. Swart et al. ** [**69**]	–	Ø	Ø	Ø	Ø	Ø	Ø	Ø	n.i.
**15. Montebarocci et al. ** [**68**]	–	x	x	x	X	x	x	n.i.	X
**Number of studies that found a deficit/number of studies that assessed this emotion**	10/12	4/11	3/11	5/11	3/11	3/11	3/11	0/1	2/6

*Note*. ‘–’ = deficits; ‘x’ = no deficits; ‘n.i.’ = not investigated; ‘Ø’ = investigated but not assessed.

### Physiological arousal

Few studies have focused on the links between the decoding of EFEs and physiological arousal at both cardiovascular and electrodermal levels. The authors of these studies assumed that greater heart rate deceleration or skin conductance activity are linked with approach or attention to meaningful stimuli (e.g., threatening [31]; arousing [135]). It has also been argued that emotional responses to EFEs refer to the simulation of the emotional state of the other [135] or to emotional empathy [136]. These authors suggest that relative to neutral expressions, positive and negative EFEs would increase skin conductance responses and would be linked with greater heart rate deceleration in response to angry faces. This might be relevant for alexithymia as it is linked with altered physiological responses to emotional stimuli and with impaired decoding of EFEs. Due to poorer simulation, lower physiological reactivity to EFEs might partly account for their altered emotional empathy and identification of EFEs. However, the studies that investigated the physiological responses to angry, happy and neutral expressions (independently of alexithymia) showed inconsistent results: some showed that angry and happy expressions trigger similar electrodermal responses (e.g., [137]) while other show stronger responses with happy than with angry expressions [31], [135]. Relative to cardiovascular responses, some studies showed heart rate deceleration in response to angry faces and acceleration to happy faces (e.g., [31]), or no difference between them [135], [137]. Because of these inconsistencies, no conclusion can be drawn from these preliminary studies. Rather, they may reveal the influence of moderators. de Sousa et al. [136] showed that empathy (measured with the BEES) moderated the skin conductance responses to angry and happy faces. They showed in control subjects with low empathy scores lower skin conductance response during the presentation of happy relative to angry faces, and no differences among high empathy scorers. This suggests that the constructs related to emotional processing may influence the physiological responses to EFEs. Alexithymia may be one of these moderators, which emphasizes the necessity to investigate the physiological responses to EFEs.

At a later stage, people have to be able to interpret these responses in order to adequately use them. The anterior insula has been critically involved in interoceptive awareness [138] and the conscious representation of internal bodily states. The level of activation in the anterior insula is positively correlated with reports of greater empathic concern for someone experiencing pain [139]. Thus, at an intrapersonal level, less interoceptive awareness may lead to an impaired understanding of one's own emotional experiences. At an interpersonal level, less interoceptive awareness might lead to poorer interpretation of the affective state resulting from the simulation of the other's emotional state. Therefore, because HA have lower activation in the anterior insula as well as lower interoceptive awareness [140], this may account for their intrapersonal deficits but also for their interpersonal deficits in terms of low EFEs decoding abilities. Furthermore, the ACC is involved in generating autonomic arousal changes. More specifically, it has an influence on autonomic control [141]. Changes in electrodermal arousal have been found to be positively correlated with ACC during a gambling task [142] and is impaired in patients with ACC lesions [143]. Therefore, we may speculate that HA's altered activation of the ACC during EFEs processing is associated with lower physiological responses to EFEs. Together with lower interoceptive awareness, lower physiological reactivity may decrease HA's opportunity to use their physiological responses as a mean to accurately decode EFEs.

### Limitations of the studies reviewed

The design inconsistencies across experiments sometimes make it difficult to directly compare studies that have investigated the relationship between alexithymia and the processing of EFE. One inconsistency is related to the type of alexithymia questionnaires used. On this issue several points may be addressed. First, it has been recommended that alexithymia, like other personality constructs, should be evaluated as a continuous variable [144]. However, this was not the case in most studies we reviewed [66], [67], [70], [71], [95]. Furthermore, alexithymia TAS scores were not dichotomized in the same way in all studies despite the cutoff recommended by Taylor et al. [20]. Second, three different auto-evaluative questionnaires were used among the studies reviewed, and sometimes without considering all the sub factors. The few available studies that considered the alexithymia subfactors seem to point to a deficit mainly for DIF and DDF. Finally, we may question the appropriateness of using auto-evaluative questionnaires to assess difficulties in processing emotional states among people who have difficulty in thinking about their emotional states. To address this problem, futures studies could use both self- and observer-evaluations (e.g., TSIA [145]; OAS [146]).

With respect to other methodological issues, some behavioural studies presented a very limited number of stimuli (e.g. : 1 picture [55]; 2 pictures [67], [70]), which is likely to compromise the reliability and generalizability of the corresponding findings. More broadly, although five studies focused on labelling abilities, there were only two studies that focused on detection and matching. Thus, the heterogeneity of the alexithymia questionnaires, the lack of studies focusing on detection and matching, and the methodological weaknesses of some studies prevent us from making strong conclusions about emotional processing deficits among HA, mainly at a behavioural level. Instead, this review reveals that other studies are needed to disentangle inconsistencies in the literature and to clarify certain questions, such as the exact nature of the perceptual processing deficits for EFEs in HA, and whether their semantic understanding of emotions is impaired.

Potential limitations also included the difficulty to have access to non-published data, which could have biased the present review. The validity of the review depends on the method of the inclusion of the studies, and thus on the publication bias, defined as the “*tendency on the parts of investigators, reviewers, and editors to submit or accept manuscripts for publication based on the direction or strength of the study findings*” ([147], p. 1385). The difficult access to non significant data are thus influenced by the fact that only the significant studies that go in the right direction are most likely to be published. This possible limitation of the present review should be kept in mind when generalizing the results to the population. In order to integrate published and non published findings, and to measure the strength of the association between alexithymia and EFEs decoding abilities (i.e,, effect size), we recommend in the future to conduct a meta-analysis.

### Directions for future research

Taken together, the data show that alexithymia may be associated with the need for more time and more information to detect, match and extract information about emotional states from facial expressions. Alexithymia may also be related to abnormal attention allocation during the visual exploration of facial expressions. Although other studies are needed to explore in depth these issues, these peculiarities in EFEs processing may account for the fact that alexithymia is associated with a lack of socio-affective skills in various comorbid disorders.

To test whether HA need more time to process EFEs [65], [73], one could manipulate the presentation time of EFEs. If HA require more time than LA to process EFEs, deficits would be revealed only under a certain time threshold. Based on the existing literature, we predict that the threshold would be around 33 msec to accurately match EFEs [65] and 300 msec to accurately label EFEs [57].

HA may also need more perceptual information to process EFEs [59]. Thus, alexithymia may be associated with a higher threshold for detecting emotional expressions, a hypothesis that may be investigated using tasks such as the Emotional Recognition Multimorph [148]. In this task, a human face evolves gradually but continuously from neutrality to a fully expressed emotion. Participants have to press a button as soon as they identify the emotion expressed by the face. If HA need more information to correctly recognize EFEs, they may take longer than LA to correctly recognize EFEs.

In order to clarify how HA allocate attention to facial features when processing EFEs, it would be important to examine the visual “scanpath” of eye movements and foveal eye fixations of HA when processing both emotional and neutral faces, using an eye-tracking procedure. It may indeed be that alexithymia is mainly associated with deficits in low level processing, such as perceptual exploration.

Furthermore, possible deficits in the semantic understanding of emotion concepts in HA should also be investigated and only one study has addressed this question in relation to the processing of EFEs [71], [72]. Moreover, only one study assessed the role of verbal abilities in the processing of EFEs [68]. Examining the relation between alexithymia and semantic knowledge of emotions may also allow us to understand why HA have worse memory for emotional words, but no memory deficit for neutral information (e.g., [149], [150]). We suggest that their poorer representations of emotion concepts and deficits in processing emotional material mutually influence each other. Poorer decoding of EFEs displayed in specific situations prevents HA to learn about the causes and more broadly the context of the arising of an emotion. Lower semantic representations of emotional concepts might reduce the attentional capture of EFEs and/or the meaning HA may attribute to the expression changes on the faces.

Relative to HA's social deficits, we know that he recognition of EFEs is fundamental to the experience of empathy (e.g., [18], [19]). Empathy requires the identification of others' EFEs and the affective experience of a similar emotion that the other. The present review suggests that additionally to behavioural deficits, several structures involved in mentalizing (i.e., identifying other's mental state) and empathizing (i.e., affective sharing), and thus in prosociality are less activated in HA relative to LA (e.g., insula, MPFC, amygdala, ACC and human mirror neurons [151] [152]). This supports the hypothesis that alexithymia might be less able to identify others' mental states and to empathize with them. Nevertheless, it remains fundamental to investigate in the future the impact of EFEs decoding deficits among HA on broader social skills (e.g., empathy; altruism).

## Conclusion

There is evidence from the studies reviewed here to support the hypothesis that alexithymia accounts for EFE labelling deficits found in some clinical disorders. Previous studies show that the confounds of depression and anxiety can account for the impairments in processing EFE seen in HA deficits to process EFEs, but only partially. Furthermore, behavioural and neuroimaging studies suggest that alexithymia is associated with processing deficits already at the perceptual level. More precisely, HA may be particularly impaired to process EFEs when these are presented briefly and/or when the image is degraded. Impoverished semantic representations of emotion concepts may also account for EFE processing deficits in HA, but future studies are needed to support this hypothesis. Finally, the results of previous studies do not allow us to determine whether alexithymia is associated with impairments in processing EFEs of a specific emotion or valence.

## Supporting Information

PRISMA Checklist S1 (DOC)

PRISMA Flow Diagram S1 (TIF)
