# Supplementary figures and images for: Alexithymia and the Processing of Emotional Facial Expressions (EFEs): Systematic Review, Unanswered Questions and Further Perspectives
Source: PLoS One. 2012 Aug 23;7(8):e42429. doi: 10.1371/journal.pone.0042429 (PMC3426527; doi:10.1371/journal.pone.0042429)

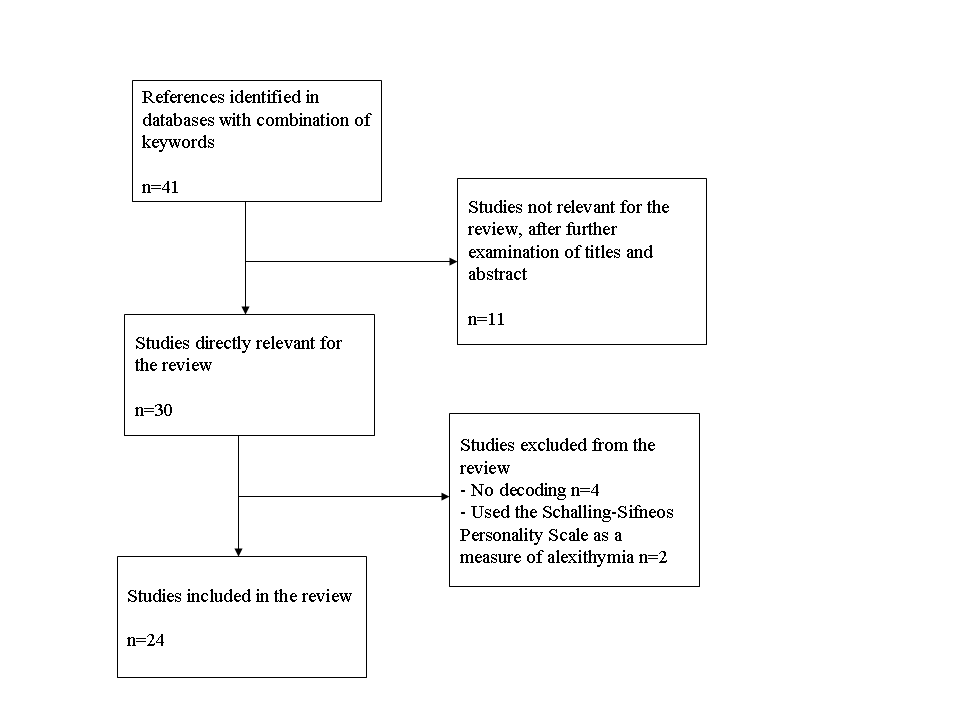

Supplement: PRISMA Flow Diagram S1 — (TIF) [file pone.0042429.s002.tif]
